# Trends in Annual Incidence Rates of Newly Diagnosed Endomyocardial Fibrosis Cases at the Uganda Heart Institute: A 14-Year Review

**DOI:** 10.3389/fcvm.2022.841346

**Published:** 2022-04-15

**Authors:** Twalib Olega Aliku, Joselyn Rwebembera, Sulaiman Lubega, Wanzhu Zhang, Charles Lugero, Judith Namuyonga, John O. O. Omagino, Emmy Okello, Peter Solomon Lwabi

**Affiliations:** ^1^Uganda Heart Institute, Mulago Hospital Complex, Kampala, Uganda; ^2^Uganda Christian University School of Medicine, Mukono, Uganda; ^3^Makerere University College of Health Sciences, Kampala, Uganda

**Keywords:** endomyocardial fibrosis, trends, incidence rates, Uganda Heart Institute, Uganda

## Abstract

**Background:**

First described in Uganda over seven decades ago, Endomyocardial fibrosis (EMF) is a rare form of restrictive cardiomyopathy found in the tropics. EMF occurs mainly in two phenotypes; biventricular involvement and right ventricular (RV) form. Previously endemic in several countries, there are reports suggesting that the disease is on the decline.

**Objectives:**

To describe trends in annual incidence rates of newly diagnosed EMF cases at the Uganda Heart Institute (UHI).

**Methods:**

This was a retrospective chart review of all newly diagnosed EMF cases at UHI from January 2007 to December 2020. Cases were divided into two groups A (2007–2013) and B (2014–2020).

**Results:**

A total of 155 cases were diagnosed during the period (Group A, *n* = 124; Group B, *n* = 31). There were no significant differences between the two groups A and B regarding median age at diagnosis (14 vs. 12 years, *p* = 0.0940), gender (48.4% female vs. 35.5%, *p* = 0.1987), and EMF type (66.9% RV EMF vs. 71.0%, *p* = 0.6634), respectively. The presence of complications such as intracardiac thrombus (5.6 vs. 32.2%, *p* = 0.0002) and pericardial effusion (57.3% vs. 80.6, *p* = 0.0172) were more frequent in group B than A, respectively. Pulmonary hypertension (PHT) was predominantly seen in cases with biventricular EMF compared to those with RV EMF (26 vs. 3.8%, *p* = 0.0001). The number of new cases diagnosed per year remained largely stable in the period 2007–2011, ranging 14–21 per year, peaked in 2012 (26 new cases), and thereafter declined from 10 cases seen in 2013 to 1–5 cases seen per year in the period 2017–2020. Similarly, the annual incidence rates of new EMF diagnosis remained relatively stable in the period 2007–2012, ranging between 22.7 and 29.7 per 10,000 patients seen in the echo labs, and then dramatically declined after 2012 to range between 1.0 and 4.5 new cases per 10,000 patients in the period between 2017 and 2020.

**Conclusion:**

There has been a steady decline in the number of new cases of EMF seen at the UHI. However, there were no significant differences in the gender, age at diagnosis and EMF subtype of cases during the period under review. Complication rates were more frequent in the later cohort.

## Introduction

Endomyocardial fibrosis (EMF) is the most common form of restrictive cardiomyopathy globally. EMF has a unique geographical distribution, with a majority of cases clustered in the tropical areas of Africa, Asia and Latin America. More than half of the cases described in literature have been reported from sub-Saharan Africa. In endemic areas, it mainly affects impoverished children and young adults, causing premature cardiovascular death. The etiologic basis and pathogenetic mechanisms involved in this disease remain unclear, with environmental, socioeconomic and genetic determinants thought to play a role (1–4).

**Figure 1:**
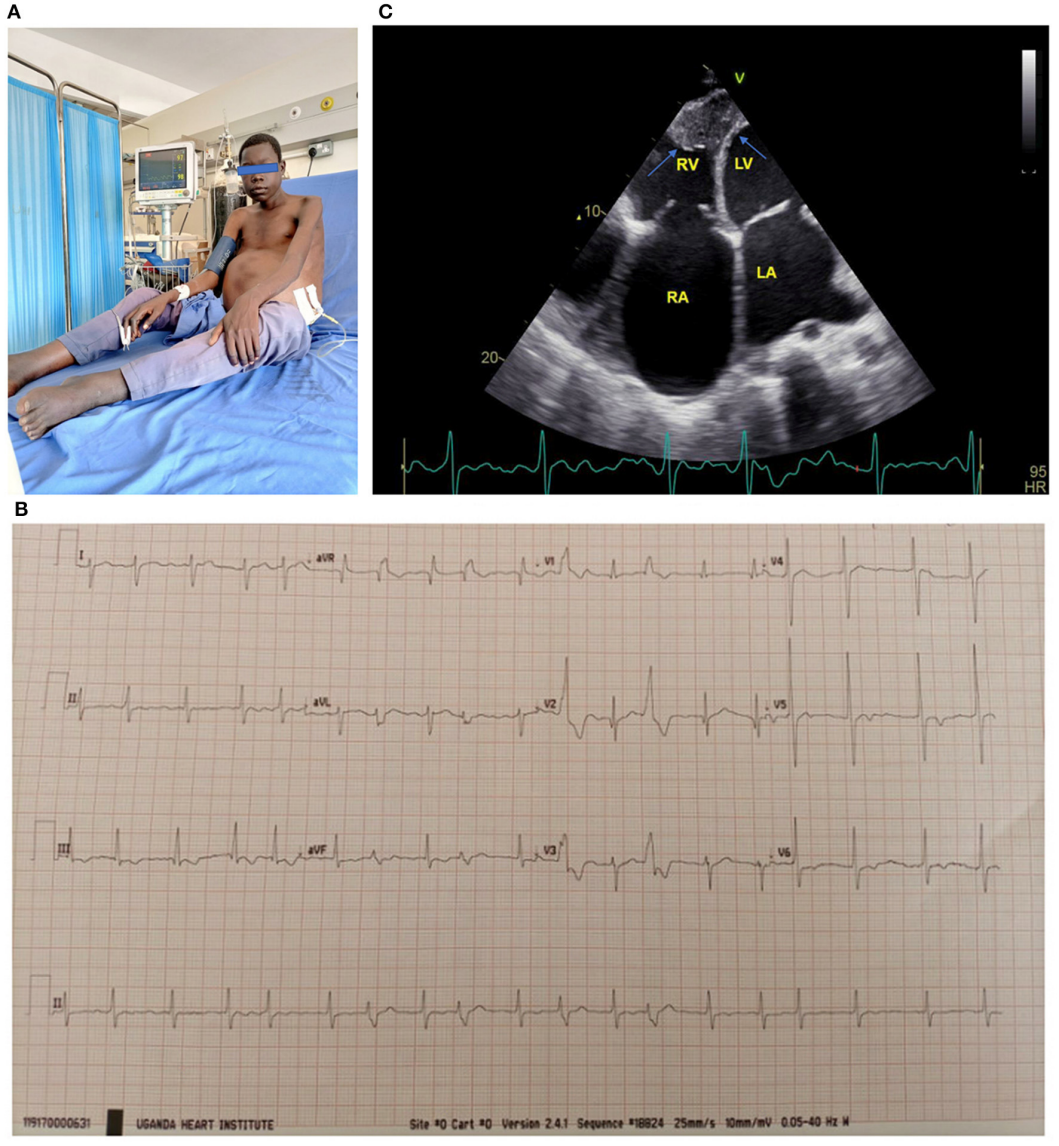
**(A)** 17-year-old boy with biventricular EMF receiving paracentesis. Note the gross abdominal ascites. **(B)** ECG of a 17-year-old boy with biventricular EMF. Note the atrial fibrillation with rapid ventricular response and frequent premature ventricular contractions. **(C)** Apical 4 chamber view of a 17-year-old boy with biventricular EMF, with predominant right ventricular involvement. Note reduced RV cavity with near obliteration of the apex and fibrotic endocardium (blue arrows) and markedly dilated right atrium. The patient's ECG tracing shows he is in atrial fibrillation. RA, right atrium; RV, right ventricle; LA, left atrium; LV, left ventricle. See the Supplementary Material for more echo images of the above patient.

The first description of EMF was made by Arthur Williams in 1938 when he found large patches of endocardial fibrosis at autopsy in two hearts in Uganda (5). Later in 1947 Bedford and Konstam described similar findings in African troops (6). It was Jack Davies who coined the term EMF as a distinct pathological disease in 1948 when he correlated typical pathological findings at autopsy with clinical features (7). The pathologic hallmark of EMF is focal or diffuse endocardial thickening caused by acellular fibro-collagen tissue deposition beneath the endothelial layer, typically affecting the ventricular apices and atrioventricular valves leading to varying degrees of valve regurgitation with resultant heart failure (8). Both acute and chronic phases are recognized in the evolution of EMF. In the acute phase, skin pruritus, hives, fever, periorbital edema and dyspnea have been described (4). Typical clinical findings in the chronic phase of EMF include stunted growth, cachexia, parotid enlargement, clubbing, hepatosplenomegaly, gross ascites with minimal pedal edema, male feminization and serum hypoalbuminemia (1, 4), (see Figure 1A).

Echocardiography is the gold standard for the diagnostic evaluation and staging of EMF. The disease occurs in three major forms: biventricular involvement (representing nearly 55% of cases), followed by predominant right ventricular involvement and then very rarely pure left ventricular involvement (9). Even in cases with biventricular involvement, the RV is usually more severely affected than the left ventricle. Endocardial thickening of the ventricles and atrioventricular valves appears as increased echo brightness of the ventricles, and results into restrictive physiology and variable degrees of mitral and or tricuspid regurgitation. Other typical findings on echocardiography include massive atrial enlargement, free or intramural thrombi, ventricular retraction with cavity obliteration, pulmonary hypertension and large pericardial effusions (4, 9), (see Figures 1B,C).

In advanced stages, EMF carries a dismal prognosis. Medical therapy is directed to treatment of heart failure symptoms and repeated pericardiocentesis or paracentesis are often performed for temporary relief. In unoperated cases, mortality typically results from progressive heart failure, arrhythmias and thromboembolism (1). While surgery improves survival and quality of life, compared with medical therapy it carries a high operative risk and is virtually inaccessible to the vast majority of those affected by the disease (1, 10–12). Some recent reports in the past decade suggest that there is a decline in the prevalence of EMF in once endemic countries (13, 14). Although the reasons for this decline are not clear, it may be related to improvements in socioeconomic status (15). The aim of this study was to describe the trends in the number of cases of EMF diagnosed at the Uganda Heart Institute (UHI) in contemporary times.

## Methods

### Study Design

This was retrospective chart review of all patients diagnosed with EMF at the UHI from January 2007 to December 2020. Data was collected from the echocardiographic reports, outpatient register, and in-patient files.

### Study Site

The UHI is the only public specialized tertiary center for cardiovascular care in Uganda. It is located within the Mulago Hospital Complex in the capital city, Kampala. The Mulago Hospital Complex serves as the country's national referral hospital and as a teaching hospital for the Makerere University College of Health Sciences. The cardiology service at the UHI serves ~14,000 patients per year. While a number of private hospitals within the capital offer echocardiography services, patients with EMF who majorly come from poor backgrounds usually seek medical services at the UHI where costs are subsidized. Patients referred to the UHI are either referred specifically for echocardiography (echo) diagnosis, in which case they would return to the primary physician/ health facility for continuity of care; or for both cardiology consultation and echo diagnosis (in which case they would subsequently be followed up at the UHI). Open heart surgery for congenital heart disease at UHI commenced in 2007 with support from visiting teams. To facilitate this, an excel sheet-based echo registry was created at the division of Pediatric cardiology of UHI in which data including patients address and contact details were captured. This registry was further expanded in 2010 and a data entrant was hired to oversee it. The division of adult cardiology maintains a word-based register of all patients seen in the echo lab. All echo reports from the UHI are archived electronically. This study was approved by the research committee of UHI.

### Study Population

All patients with diagnosis of EMF were included into the study. Each patient was counted only once at the date of first echocardiography exam, and the echocardiographic report at initial presentation was used for data capture. Since the echo reports at UHI routinely don't have patients' physical address information, this data was obtained from the outpatient/inpatient visit forms. Cases were divided into two groups: Group A (cohort diagnosed between 2007 and 2013) and Group B (diagnosed in the period 2014–2020). The year 2014 was used as demarcation between the two cohorts to allow for comparison of two equal time spans within the study period.

### Echocardiography Procedures and Diagnosis of EMF

All transthoracic echocardiograms (TTE) were performed by pediatric or adult cardiologists in accordance with established guidelines (16–18). The diagnosis of EMF was based on typical echocardiographic features that have been previously published (8, 9). No endocardial biopsies were done to confirm the echo diagnosis. No patient underwent cardiac magnetic resonance (CMR) imaging as this service was not available in the country during the study period.

### Statistical Analysis

Data analysis was performed using MedCalc^®^. Continuous data is presented as means, medians (1QR) and percentages. Annual incidence rates of newly diagnosed EMF were calculated as number of new cases of EMF per 10,000 patients seen in the echo labs. Median ages were compared using Mann-Whitney test. Differences in proportions were analyzed using the χ2-test or Fisher's exact test as appropriate and *p* < 0.05 were considered statistically significant.

## Results

**Figure 2:**
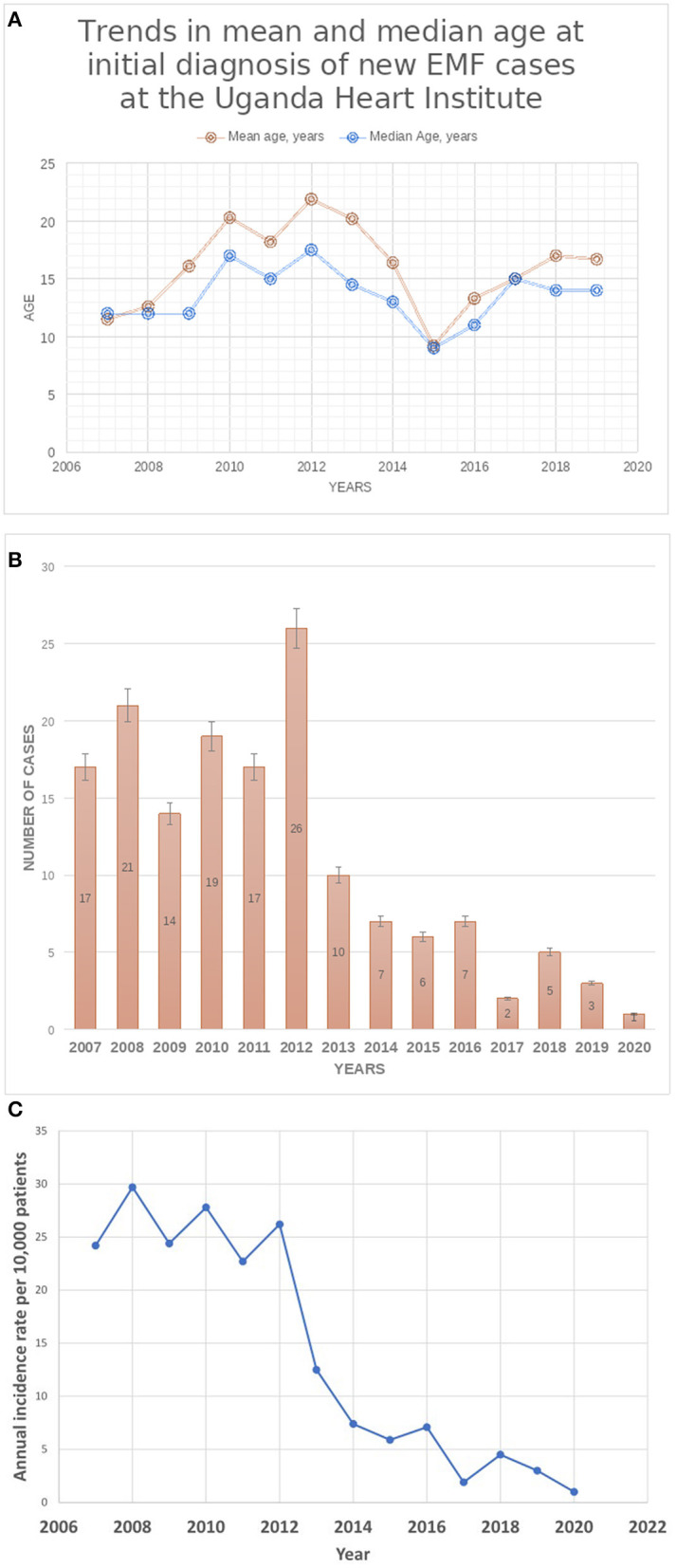
**(A)** Trends in mean and median age at initial diagnosis of new EMF cases at UHI. **(B)** Trends in Total Number of New cases of EMF diagnosed at the Uganda Heart Institute. **(C)** Trends in the annual Incidence of rates of newly diagnosed EMF at the Uganda Heart Institute. Note the dramatic decline in the incidence rates after the year 2012.

A total of 155 cases were seen during the period (Group A, *n* = 124; Group B, *n* = 31). The median age at initial diagnosis was 13 years (IQR:10–18). Females comprised 45.8% of cases (*n* = 71). The predominant EMF phenotype was right ventricular EMF, accounting for 67.7% (*n* = 105) of cases, with the rest of cases having bi-ventricular EMF (*n* = 50, 32.3%), (see Table 1). No cases of pure left ventricular EMF were seen. The majority of cases with biventricular EMF had predominant RV involvement (*n* = 42, 84%) with the rest (*n* = 8, 16%) having equal involvement of both right and left ventricles. There were no significant differences between the two groups A and B regarding median age at diagnosis (14 vs. 12 years, *p* = 0.0940), gender distribution (48.4% female vs. 35.5%, *p* = 0.1987), and EMF type (66.9% RV EMF vs. 71.0%, *p* = 0.6634) respectively, (see Table 1). However, we noted that over the years, both the mean and median age at diagnosis progressively increased over the course of the study period (see Figure 2A). The youngest patient was a 16-month-old girl with RV EMF. The oldest patient was an 80-year-old woman with RV EMF. Children (age <18 years) comprised 73.5% (*n* = 114) of cases.

**Table 1:** Showing baseline characteristics of EMF cases.

**Variable**	**Overall *N* = 155**	**Group A *N* = 124**	**Group B *N* = 31**	**P-value**
Median age in years (1QR)	13 (10–18)	14 (10.5–19.0)	12 (10.0–14.75)	0.0940
Female gender, *n* (%)	71 (45.8)	60 (48.4)	11 (35.5)	0.1987
EMF phenotype				
Right ventricular EMF, *n* (%)	105 (67.7)	83 (66.9)	22 (71.0)	0.6634
Biventricular EMF, *n* (%)	50 (32.3)	41 (33.1)	9 (29.0)	

*No cases of pure left ventricular EMF were seen. The majority of cases with biventricular EMF had predominant RV involvement (n = 42, 84%) with the rest (n = 8, 16%) have equal involvement of both right and left ventricles*.

Significant atrioventricular valve regurgitation (AVVR), defined as moderate or severe regurgitation of either tricuspid or mitral valves was found in 87.7% of cases (*n* = 136), while 62.3% (*n* = 96) of cases had pericardial effusion, (see Table 2). Intracardiac thrombi were found in 11% (*n* = 17) of cases, all of which were located in the right atrium except in one case where additionally there was a thrombus in the right ventricular outflow tract. Pulmonary hypertension (defined as tricuspid valve regurgitant jet gradient >35 mmHg) was present in 11% of cases (*n* = 17). When comparing the two cohorts A and B respectively, presence of intracardiac thrombi (5.6 vs. 32.2%, *p* = 0.0002) and pericardial effusion (57.3% vs. 80.2, *p* = 0.0172) were significantly less frequent in group A than B. There were no significant differences in the frequencies of pulmonary hypertension (11.3 vs. 9.7%, *p* = 1.000) and presence of significant AVVR (87.9 vs. 87.1%, *p* = 0.9036) between the two groups A and B respectively.

**Table 2:** Comparison of selected echocardiographic features of the two cohorts of cases.

**Variable**	**Overall *N* = 155**	**Group A *N* = 124**	**Group B *N* = 31**	**P-value**
Intracardiac thrombus	17 (11.0)	7 (5.6)	10 (32.2)	0.0002
Spontaneous echo contrast	19 (12.3)	14 (11.3)	5 (16.2)	0.5396
Intracardiac thrombus or	36 (23.4)	21 (16.9)	15 (48.4)	0.0005
spontaneous echo contrast^**^				
Pericardial effusion present	96 (62.3)	71 (57.3)	25 (80.6)	0.0172
Significant* AVVR	136 (87.7)	109 (87.9)	27 (87.1)	0.9036
Significant MR	28 (18.2)	23 (18.5)	5 (16.1)	1.0000
Pulmonary hypertension present,	17 (11.0)	14 (11.3)	3 (9.7)	1.0000
*n* (%)				

** *Spontaneous echo contrast without intracardiac thrombus*.

*Significant AVVR was defined as moderate or severe regurgitation of either tricuspid or mitral valves. AVVR, Atrioventricular valve regurgitation; MR, Mitral regurgitation*.

Pulmonary hypertension (PHT) was predominantly seen in cases with biventricular EMF compared to cases with RV EMF (26.0 vs. 3.8%, *p* = 0.0001), (see Table 3). On the contrary, the presence of pericardial effusion was significantly more frequent in cases with RV EMF compared to cases with biventricular EMF (66.8 vs. 48.0%, *p* = 0.0138). There were no significant differences in median age (13 vs. 14 years, *p* = 0.4040), gender (45.7 vs. 46.0 % female*, p* = 0.9721) and frequency of intracardiac thrombi (12.4 vs. 8.0%, *p* = 0.5481) between cases with right ventricular and biventricular EMF phenotypes, respectively.

**Table 3:** Comparison of cases with right ventricular EMF vs. those with biventricular EMF.

**Variable**	**Right ventricular EMF, *N* = 105**	**Biventricular EMF, *N* = 50**	**P-value**
Median Age in Years, IQR	13 (10–17)	14 (11–24)	0.4040
Female gender *N* (%)	48 (45.7)	23 (46.0)	0.9721
Intracardiac thrombus *N* (%)	13 (12.4)	4 (8.0)	0.5841
Spontaneous echo contrast	16 (15.2)	3 (6.0)	0.1022
Spontaneous echo contrast or	29 (27.6)	7 (14.0)	0.0614
intracardiac thrombus			
Pericardial effusion *N* (%)	72 (68.6)	24 (48.0)	0.0138
Pulmonary Hypertension, *N* (%)	4 (3.8)	13 (26.0)	0.0001

The number of new cases of EMF diagnosed per year at the UHI remained largely stable in the period 2007–2011, ranging from 14 to 21 per year, peaked in 2012 (26 new cases), and thereafter declined from 10 cases seen in 2013 to 1–5 cases seen per year in the period 2017–2020, (see Figure 2B). Similarly, the annual incidence rates of new EMF diagnosis remained relatively stable in the period 2007–2012, ranging between 22.7 and 29.7 per 10,000 patients seen in the echo labs, and then dramatically declined after 2012 to range between 1.0 and 4.5 new cases per 10,000 patients in the period between 2017 and 2020 (see Figure 2C). The number of patients seen in the echo labs and total in-patient admissions per year at UHI increased over the course of the study period (see Supplementary Table 1).

**Figure 3:**
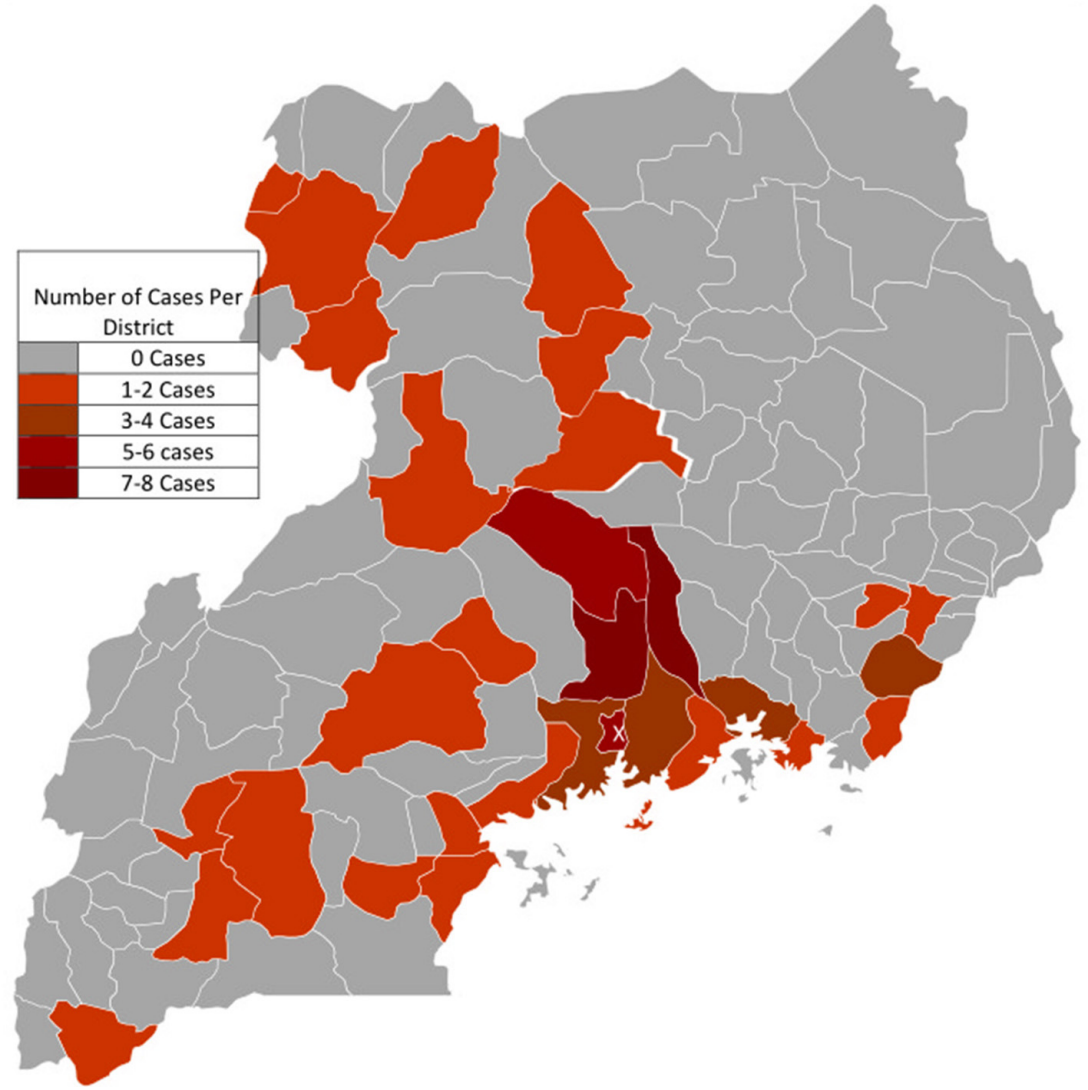
Geographic Origin of Cases with Endomyocardial fibrosis. X: The capital City Kampala where The Uganda Heart Institute is located in the Mulago National Specialized Hospital Complex. Cases were mostly clustered around districts bordering Kampala, especially to the East and North of Kampala.

Data on the district of origin was available in 45.8% of cases (*n* = 71). In total, cases originated from 32 out of 135 (23.7%) districts of Uganda. Cases of EMF originated mainly from the districts found in the central region of Uganda. The majority of cases were clustered in the districts surrounding Kampala, majorly to the East and North of the city (see Figure 3), with Kayunga and Luwero districts leading with 8 cases each, followed by both Kampala (*n* = 6) and Nakasongola (*n* = 5). Few cases were registered from parts of northern and western Uganda. No cases were registered from the North Eastern parts of the country.

In this study three patients with advanced disease received surgery (two locally at UHI with a visiting surgical team and one abroad) in 2012, but all had early in-hospital mortality. Since then, no surgeries have been attempted at the UHI.

## Discussion

Whereas, cases of EMF have been described in several parts of Africa, in part facilitated by increasing access to accurate diagnostic capability (19), there is paucity of robust data on the epidemiological trends of disease burden in affected countries. In this paper we report for the very first time the declining trends in the annual incidence rates of new cases of EMF seen at UHI, the major national referral facility for cardiovascular care in Uganda, a country where the disease was first described over seven decades ago and where it remained endemic for a long time. In 1998, an echocardiography study of 500 consecutive patients with heart disease seen over a 10-month period in the department of Medicine of Mulago hospital, found EMF to be the commonest cardiac abnormality seen, accounting for 19.8%(*n* = 99) of cases (20). Among 826 cases of acquired heart disease among children aged <15 years seen at the UHI between January 2007 to December 2011 (21), EMF was found to be the fourth major cause at 7.1% of cases (*n* = 59) after rheumatic heart disease (45.5%), dilated cardiomyopathy (22.4%), pericarditis (8.8%) and cor pulmonale (7.9%). This observation of declining trends in the number of EMF cases is in tandem with what has been previously described in countries such as Nigeria and the Southern Indian state of Kerala (13, 14, 22). In a retrospective review of 7,956 cardiac patients attending a university teaching hospital in Nigeria between 2003 and 2009, only three cases of EMF were recorded (accounting for only 0.04% of cardiac patients), compared with a prevalence of 10% in the same country during the era between 1960's and 1970's (13).

Predominant right ventricular involvement was the commonest EMF phenotype seen in our series, accounting for nearly two thirds of cases. This is similar to what has been described previously (23). We did not find significant differences in age at diagnosis, gender distribution and EMF phenotype in between the two groups representing two time periods of equal span. However, we noted that there was a trend toward older mean and median age of patients over the course of the years compared to that at the beginning of the study period. In 2009, Tharakan and Bohora (22) reported dramatic declines in numbers of patients with new diagnosis of EMF over the course of the previous two decades. They also observed an epidemiologic transition in the presentation of the disease, where patients in the contemporary era were noted to be much older, less symptomatic, with less severe disease, often diagnosed when being evaluated for non-specific symptoms and had excellent medium-term prognosis. They proposed that the demographic change to higher age at diagnosis may be attributable to an absolute decrease in the incidence of the disease and patient presentation with milder forms of EMF later in life. It is also possible that the intensity of putative environmental factors in the causal pathway of EMF lessens to a significant degree so as to result in declines in incidence rates and disease severity.

In this study, we did observe some differences in disease presentation over the course of the study period. The proportions of patients with different EMF phenotypes, the severity of atrioventricular valve regurgitation and the presence of pericardial effusion did not significantly differ between the two study periods. However, the rate of complications such as the presence of intracardiac thrombus and pericardial effusion were significantly more common in the later cohort than the first, suggesting later diagnosis of the disease (4, 8). This unexpected finding may be explained by the fact that as disease incidence rates decline, it declines in the priority list of possible differential diagnoses made by health care providers, causing delays in patient referral for diagnostic evaluation, and hence presentation with advanced disease. While access to centers with high quality echocardiographic services has slightly improved in the country within the past half decade, these are only located mainly in a few selected cities in the central, mid-eastern and western parts of the country.

The geographic origin of EMF cases seen at Mulago National Referral and Teaching hospital was previously found to be clustered in the districts of Mukono and Luwero, which neighbor Kampala, the capital of Uganda. The disease was noted to affect predominantly peasant migrants of Rwandese/ Burundian origin and the indigenous Baganda (the most populous tribal group in Uganda) in these areas living in conditions of poverty (20, 24, 25). Similarly in this study, districts with the highest number of EMF cases were Kayunga (which was curved out of the district of Mukono in the year 2000), and Luwero, each accounting for eight (8) cases, followed by the districts of Kampala (*n* = 6), and Nakasongola (*n* = 5) which was also created from the district of Luwero in 1997. Luwero accounted for four (4) cases. There were no cases from districts in the North Eastern region of Uganda. It is worth noting that after several decades, cases of EMF still predominantly **originate** from the same geographical areas. The clustering of most cases from districts around the capital Kampala, where the UHI is located could also be attributable to easier access to diagnostic facilities. Geographical clustering of EMF cases to particular areas, as described in several studies (9, 24–26), may suggest that the potential cause of this disease is highly specific and localized (27), and hence probably environmental in origin. Ferreira et al. (26) report in a retrospective review of 118 EMF cases from three provinces of southern Mozambique treated at the Maputo Central Hospital between 1987 and 1999, nearly two thirds of the patients (*n* = 77) came from the Inhambane province and all cases were native to districts located in the costal region. Most of the cases of EMF seen in the present study were clustered in districts bordering Lake Victoria, the Victorian and Albert Nile, suggesting a possible aetiologic link with hepatosplenic schistosomiasis that is common in areas near water bodies. The association between schistosomiasis and EMF has been previously suggested, (19, 28, 29), but these have not yet been proven. The biologic plausibility for such an association is considered sound since both advanced EMF and schistosomiasis share certain typical clinical features like cachexia, hepatosplenomegaly and gross ascites with minimal peripheral edema (19). In this study we did not examine the tribal origin of EMF cases.

The diagnosis of EMF in this study was based on typical findings on transthoracic echo. Endomyocardial biopsy (EMB) often used as an additional confirmatory test when available. Echocardiographic features have been found to correlate well with histological findings from surgical and autopsy specimens (30), and hence echo is a diagnostic tool of choice in resource limited settings (19). Characteristic histological features include a fibrotic endocardium alongside variable degrees of inflammatory changes dependent on disease stage. These include inflammatory cell infiltration, neovascularization and loss of endomyocardial transition due to fibrinoid deposition in the early stages. In the later stages there is often marked endocardial thickening, hyalinization and reduced cellularity (19). CMR imaging is another useful non-invasive diagnostic tool since it permits myocardial tissue characterization and morpho-functional assessment in EMF (31, 32). In the acute phase it demonstrates the ventricular dilatation, myocardial edema, and diffuse subendocardial late gadolinium enhancement (LGE) pattern. In the chronic phase, CMR imaging can reveal basal wall thinning, apical fibrous obliteration, superimposed thrombosis, absence of myocardial edema and diffuse subendocardial LGE pattern (31). The major limitation of CMR is the lack of access which is common in the most underserved regions of the world.

The overall prognosis for patients with EMF is dismal. Medical therapy is limited to treatment of heart failure, arrhythmias and anticoagulation. High dose diuretics and repeated paracenteses are often necessary because of gross ascites for symptom relief. During this study, three (3) patients in this cohort underwent surgery, two locally and one from abroad. Immediate post-operative mortality occurred in all three cases. Surgery typically involves targeted resection of the fibrosed endocardium with valve replacement and bidirectional Glenn shunt in cases were the right ventricular volume remains suboptimal following endocardial resection. While surgical techniques have improved over the years, long-term survival following surgery remains unsatisfactory (33) and the endocardial fibrosis may recur (11). Outcomes after surgery are best when performed before advanced disease. The reasons for the dramatic declines in the incidence of EMF in once endemic hot-spots of the disease around the globe are unclear. It has been attributed in part due to improvements in living conditions and socioeconomic status of at-risk populations. Albeit slow, Uganda has also registered some improvements in human development indices, from 0.322 in 1990 to 0.544 in 2019 (34). During the study period, the country's GDP per capita (in current US dollars) doubled from $403 in 2007 to $815 in 2019 (35), and the childhood under-five mortality rate per 1,000 live births declined nearly by half from 93.5 in 2007 to 45.8 in 2019 (36). The declining trends in the incidence of EMF across the globe must cause scientists to re-examine the hypothesis in the causation of this neglected tropical disease.

This was a hospital-based study, and hence may not accurately reflect the actual disease burden present in affected communities. Patients presenting to a tertiary care facility often reflect a selected number with symptomatic disease, who have the means to navigate complex health system referral pathways common to low resource settings. There is therefore need for targeted and prospective community based epidemiological surveys to uncover the actual burden of asymptomatic EMF in the country, especially in areas of geographic clustering of cases in the districts surrounding Lake Victoria and the Nile as demonstrated in this paper. Such a study could utilize newly developed echocardiography-based disease staging algorithms as used in Mozambique (9). It could also pragmatically examine the link between schistosomiasis and EMF in Uganda.

### Limitations

This study has several limitations. Being a retrospective echocardiographic based study, it was not possible to obtain data on other pertinent demographic variables such as tribal origin of cases, socioeconomic status, functional status of patients and rhythm abnormalities. We did not collect data on laboratory findings since the majority of patients were referred to UHI as outpatients specifically for echo evaluation and cardiology consultation. As a result, we cannot report on association with eosinophilia that has been well-described. Apart from the two cases who had surgery locally, endomyocardial biopsies were not done. Data on geographic origin of cases was also incomplete.

## Conclusion

We noted declining trends in the incidence rates of EMF cases seen at the Uganda Heart Institute in the 14-year period 2007–2020. There were no significant differences in gender, age at diagnosis and EMF phenotype over the study period. Complications suggesting advanced disease such as presence of intracardiac thrombus and pericardial effusion were more frequent in patients diagnosed in the latter half of the study period.

## Data Availability Statement

The raw data supporting the conclusions of this article will be made available by the authors, upon reasonable request.

## Ethics Statement

The studies involving human participants were reviewed and approved by Uganda Heart Institute. Written informed consent from the participants' legal guardian/next of kin was not required to participate in this study in accordance with the national legislation and the institutional requirements. Written informed consent was obtained from the minor(s)' legal guardian/next of kin for the publication of any potentially identifiable images or data included in this article.

## Author Contributions

TA conceived and designed the project, collected data, performed echocardiograms, provided patient care, performed data analysis, and drafted the first version of the manuscript. JR, SL, WZ, JN, PL, CL, and EO performed echocardiograms, provided patient care, and made substantial contributions to data analysis and interpretation. JO provided patient care and administrative oversight. All authors critically reviewed the manuscript for important intellectual content and approved the final manuscript.

## Conflict of Interest

The authors declare that the research was conducted in the absence of any commercial or financial relationships that could be construed as a potential conflict of interest.

## Publisher's Note

All claims expressed in this article are solely those of the authors and do not necessarily represent those of their affiliated organizations, or those of the publisher, the editors and the reviewers. Any product that may be evaluated in this article, or claim that may be made by its manufacturer, is not guaranteed or endorsed by the publisher.

## Supplementary Material

Supplementary Video 1 Apical 4 chamber of a 17 year old male with biventricular EMF. Note near obliteration of RV apex with reduced RV cavity, endocardial thickening.

Supplementary Video 2 Color Flow Doppler image of same patient showing severe tricuspid valve regurgitation.

Supplementary Video 3 Color Flow Doppler of the same patient showing at least moderate mitral valve regurgitation.

Supplementary Table 1 Showing trends in the numbers, mean and median ages, and incidence rates of new EMF diagnosis seen at the Uganda Heart institute, alongside Uganda's GDP per capita and Under-5 mortality rates.
