# Supplementary material for: Trends in Annual Incidence Rates of Newly Diagnosed Endomyocardial Fibrosis Cases at the Uganda Heart Institute: A 14-Year Review
Source: Front Cardiovasc Med. 2022 Apr 15;9:841346. doi: 10.3389/fcvm.2022.841346 (PMC9051226; doi:10.3389/fcvm.2022.841346)
Supplement: Supplementary Table 1 — Showing trends in the numbers, mean and median ages, and incidence rates of new EMF diagnosis seen at the Uganda Heart institute, alongside Uganda's GDP per capita and Under-5 mortality rates. [file Table_1.DOCX]

| **Year/Category** | **2007** | **2008** | **2009** | **2010** | **2011** | **2012** | **2013** | **2014** | **2015** | **2016** | **2017** | **2018** | **2019** | **2020** |
| --- | --- | --- | --- | --- | --- | --- | --- | --- | --- | --- | --- | --- | --- | --- |
| Total Number of New EMF Cases | 17 | 21 | 14 | 19 | 17 | 26 | 10 | 7 | 6 | 7 | 2 | 5 | 3 | 1 |
| Mean Age of Cases | 11.5 | 12.6 | 16.1 | 20.3 | 18.2 | 21.9 | 20.2 | 16.4 | 9 | 13.3 | 15 | 17 | 16.7 | 6 |
| Median Age of cases | 12 | 12 | 12 | 17 | 15 | 17.5 | 14.5 | 13 | 9.2 | 11 | 15 | 14 | 14 | 6 |
| Total patient ECHOs per year at UHI | 7039 | 7066 | 5745 | 6830 | 7,483 | 9,920 | 8,028 | 9,450 | 10,170 | 9,880 | 10,776 | 11,141 | 9,937 | 10,345 |
| **Incidence rates of new EMF per 10,000 patient echos at UHI** | **24.2** | **29.7** | **24.4** | **27.8** | **22.7** | **26.2** | **12.5** | **7.4** | **5.9** | **7.1** | **1.9** | **4.5** | **3.0** | **1.0** |
| **Total number of inpatient admissions at UHI** | **849** | **964** | **952** | **1014** | **1219** | **1309** | **1372** | **1663** | **1696** | **1184*** | **1471** | **1534** | **1373** | **1820** |
| Uganda’s GDP per capita ** | 403 | 474 | 796 | 819 | 828 | 786 | 806 | 879 | 843 | 733 | 746 | 770 | 794 | 814 |
| Uganda’ Under-5 Mortality per 1000 live births## | 93.5 | 87.5 | 82.1 | 77.0 | 72.5 | 67.6 | 63.3 | 59.4 | 56.1 | 52.8 | 50.2 | 48 | 45.8 |  |

1. * The reduction of patient admissions in this year was due to the commencement of renovations at UHI, with the inpatient service moved to another unit within the Mulago Hospital Complex that had less bed capacity than before.
2. **GDP per capita data obtained from. GDP per capita (Current US $)-Uganda. <https://data.worldbank.org/indicator/NY.GDP.PCAP.CD?locations=UG>. Last accessed 11 August 2021
3. ## Under-5 mortality. <https://data.unicef.org/country/uga/> Last accessed 11 August 2021
